# Reconstruction of Composite Soft Tissue Defect in the Distal Finger Using Partial Toenail Flap Transfer

**DOI:** 10.1111/os.13829

**Published:** 2023-08-29

**Authors:** Leyi Cai, Xian Zhang, Yingying Zhang, Guangheng Xiang, Peng Luo, Zhijie Li, Feiya Zhou

**Affiliations:** ^1^ Department of Orthopaedic Surgery The Second Affiliated Hospital and Yuying Children's Hospital of Wenzhou Medical University Wenzhou China; ^2^ Department of Operation Care Unit The Second Affiliated Hospital and Yuying Children's Hospital of Wenzhou Medical University Wenzhou China

**Keywords:** Digitsoft Tissue Defect, Surgical Flap, Toenail Flap

## Abstract

**Objective:**

Composite tissue loss involving the distal finger pulp and the nail is a common but challenging finger injury to restore. This study introduces a reconstruction procedure for a distal finger pulp and nail defect using a partial toenail flap transfer.

**Methods:**

Twenty digits, including 16 thumbs, two index fingers, and two middle fingers, with composite soft tissue defects were treated with a partial toenail flap transfer from October 2015 to January 2020. Shortening revision of the great toe phalanx, a V‐Y advancement flap of the toe pulp, and a local pedicle flap from a second toe transfer were used to cover the donor sites, and no skin grafts were required. Functionality was evaluated using the validated Spanish version of the Quick‐DASH scale. The aesthetics of both the reconstructed and donor sites were evaluated using the Vancouver Scar Scale (VSS). The static two‐point discrimination (2‐PD) of the finger pulp was used as a measure of tactile agnosia.

**Results:**

All donor site wounds healed well. The average follow‐up time was 23.6 months (6–39 months). The mean Quick‐DASH functional score was 7.1. The VSS scores were 4.02 ± 0.29 and 4.00 ± 0.38 for the reconstructed and donor sites, respectively. The static 2‐PD of finger pulp was 4.5 ± 0.76 mm. The patients were satisfied with finger motion, sensory function, and aesthetic contour.

**Conclusions:**

Partial toenail flap transfer is the recommended treatment to regain motion, sensation, function, and a satisfactory aesthetic appearance when considering repairing a composite soft tissue distal finger defect with accompanying loss of the perionychium, particularly in the thumb, index finger, or middle finger.

## Graphical abstract

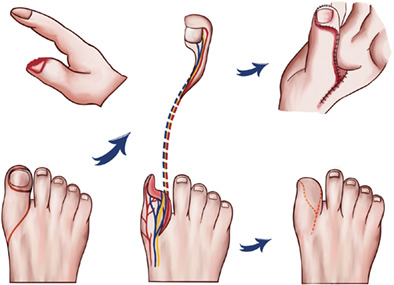

## Introduction

Fingertip loss includes loss of the distal finger pulp, the nail, the distal digit nerve, and occasionally part of the distal phalanx. The principles of reconstructing a fingertip differ greatly from those of other portions of the finger. Fingertip reconstruction requires a good tissue pad, excellent sensation, a stable well‐formed nail, and an aesthetic contour. If the anatomic requirements of fingertip reconstruction cannot be achieved, shortening the phalanx bone and revision is another method with an acceptable outcome.^1^ However, loss of finger length leads to reduced hand function and can affect a patient's confidence. Various skin resurfacing methods have been applied for this common but challenging problem. Toe tissue transfer is a unique method that can be used to anatomically restore multiple tissue‐distal finger pulp and nail loss in a single stage without further damage to the affected hand.

Toenail flap transfer is a well‐established procedure that is widely applied to reconstruct thumb and finger defects. The tissue transfer from the great toe is a reconstructive method for finger‐like restoration. Although it has been more than 40 years since the first microvascular toe‐to‐finger transfer was performed, donor site morbidity has become an increasing concern.^2^ The surgeon's ultimate goal is to balance satisfactory restoration of the recipient site and minimize donor deformity.

In this article, we want to (i) demonstrate that the partial toenail flap transfer was valuable in restoring lost tissue in one stage with minimal deformity at the donor site when a segmental distal finger tissue defect occurred in the thumb, index finger, or middle fingertip; and (ii) share the clinical outcomes using a partial toenail flap transfer to reconstruct the distal finger pulp and the perionychium to achieve a natural restoration.

## Patients and Methods

### Patients

The inclusions were (i) patients aged 18–80 years; (ii) patients who sustained a machine‐crushing injury or a cut injury with defects in the distal finger tissue; and (iii) patients who underwent at least 12 months of follow‐up. The following patients were excluded: (i) those with finger fractures; (ii) those without a great toe; and (iii) patients whose general condition was too poor to tolerate surgery.

This study was performed in line with the principles of the Declaration of Helsinki. Approval for this case‐control investigation was obtained from the Second Affiliated Hospital and Yuying Children's Hospital of Wenzhou Medical University Institutional Review Board (No. 2022‐YL‐11‐01).

### Surgical Technique

#### Preparation of the Recipient Area

The circumference of the distal finger pulp and the width of the nail bed of the contralateral healthy finger were measured before the operation. The operations were carried out under anesthesia with proper tourniquet control. The recipient site was carefully and thoroughly debrided before the flap was harvested. The health of the vessels and nerve stump was confirmed, and they were prepared for the anastomosis. The sizes of the residual nail bed and finger pulp defect were measured to outline the partial toenail flap. Bone or tendon injuries were repaired. No phalanx fracture, joint injury, or tendon rupture was observed in this group.

#### Operation of Skin Flap Transplantation

The partial toenail flap was outlined on the ipsilateral or contralateral great toe following the defect shape and the side of the defect. The length and width of the nail and other perionychium tissue were precisely marked based on the tissue size and calculated before the surgery. An S‐shaped incision was made on the dorsal side of the great toe, which cut off the required nail bed and ended proximally at the first web. The subcutaneous dorsal veins were exposed and retrograde tracked into the flaps with accurate and careful dissection. The dorsal aspect mainly consisted of the nail bed and a small amount of skin, which made it difficult to include the veins in the flap. A 1‐cm wide subcutaneous soft tissue band was retained around the vein to make the procedure safer when the dorsal toe skin was incised. After confirming that at least one dorsal digital vein entered the fascial flap, the entire vein was separated from the first web or the dorsal metatarsophalangeal and ligated.^10^ The proximal S‐shaped incision was closed in the first web, and the proper fibular plantar artery and the proper nerve of the toe were easily dissected in this incision. The appropriate lengths of the vessels and nerve were retained and cut according to the defect in the recipient area but typically not beyond the first web.

#### Surgical Coverage of the Recipient Area

**FIGURE 1:**
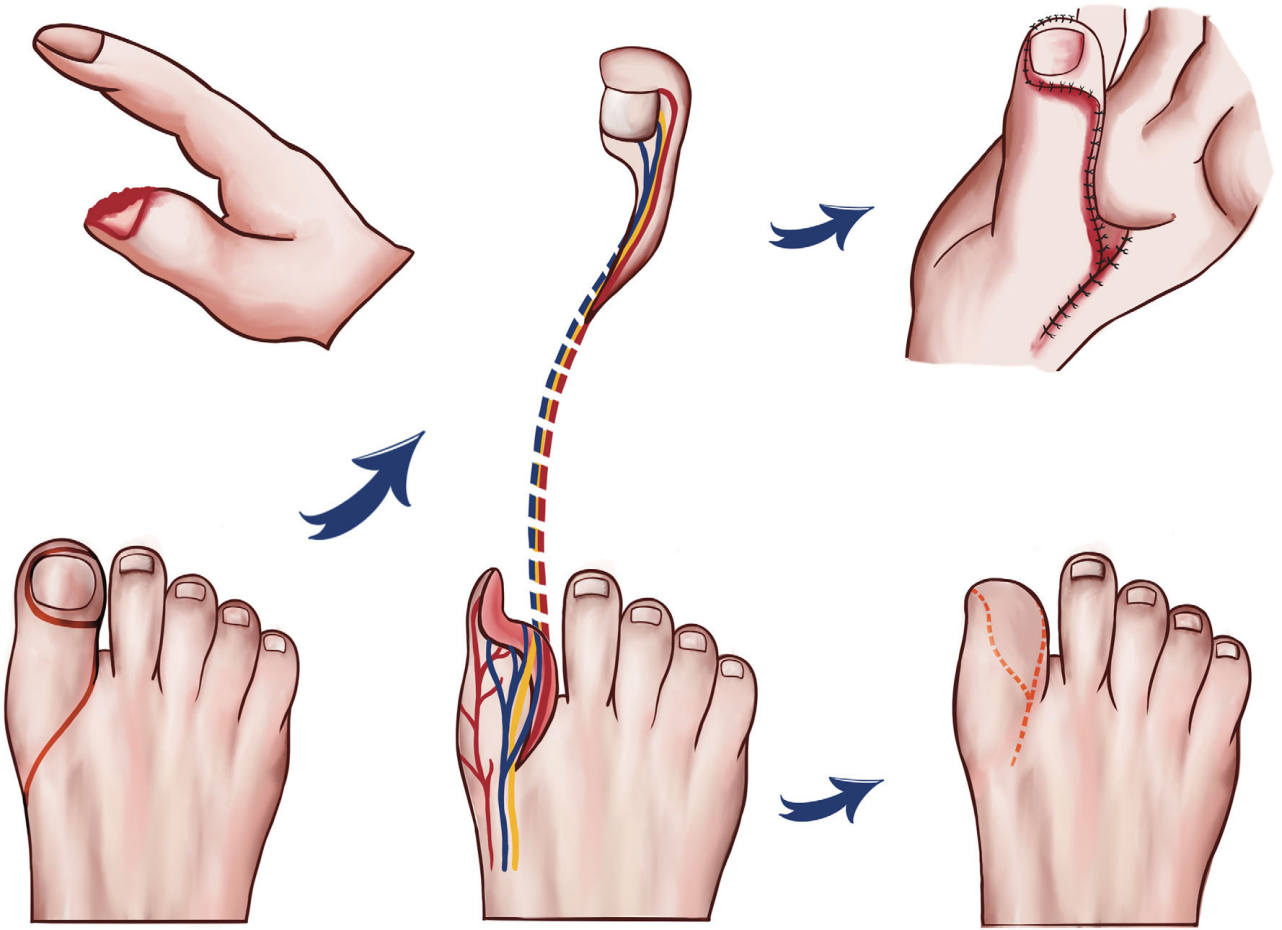
Schematic drawing demonstrating the extra triangular skin harvested around the vessel pedicle and the dorsal vein protected by the surrounding subcutaneous tissue.

The plantar artery was anastomosed with the proper digital artery, the dorsal foot cutaneous vein was anastomosed with the dorsal finger vein, and the plantar nerve was fixed with the digital nerve stump. The toe phalanx shortening revision, the toe pulp V‐Y advancement flap, and the local pedicle flap transfer from the second toe were performed to cover the donor sites (Figure 1).

### Evaluation of Outcomes

Functionality was evaluated using the validated Spanish version of the Quick‐DASH scale,^3^ which assesses upper extremity motor functions. The highest possible score is 100; higher scores reflect poorer upper limb function. The aesthetics of both the reconstructed and donor sites were evaluated using the Vancouver Scar Scale (VSS),^4^ which assessed pigmentation, vascularity, pliability, and height. The static two‐point discrimination (2‐PD)^5^ of the finger pulp was used as a measure of tactile agnosia; this indicates finger nerve recovery.

## Result

### Patient Characteristics

Twenty patients (16 thumbs, two index fingers, and two middle fingers) were treated with the partial toenail flap transfer between October 2015 and October 2020. There were 16 male and four female patients, with a mean age of 28.5 ± 8.86 years (range 18–54 years). The sizes of the flaps raised during the operation ranged from 3.0 cm × 1.5 cm to 5.5 cm × 3.5 cm. The toe phalanx shortening revision, the toe pulp V‐Y advancement flap, and the local pedicle flap transfer were performed to cover the donor sites without a skin graft. In one patient with a trauma history for his injured thumb, an end‐to‐side anastomosis of the radial artery and dorsal metatarsal artery was performed. The other patients were anastomosed with the digital artery. Fourteen donor site cases were covered with toe bone shortening and stump revision. Three cases were covered with the toe pulp V‐Y advancement flap, and three cases were covered with the second toe neurovascular pedicle flap transfer. The longest follow‐up was 3 years, and no adverse donor site effects were observed.

### Patient Outcomes

All flaps survived without complications (e.g., vascular crisis, partial necrosis, or infection) during the 6‐ to 39‐month follow‐up. The patients were satisfied with finger motion, sensory function, and aesthetic contour. The mean Quick‐DASH functional score was 7.1 (range: 4–11). Thus, the disability rate was low, and grabbing or holding a pen or glass of water with the upper limb was possible. The VSS scores were 4.02 ± 0.29 and 4.00 ± 0.38 for the reconstructed and donor sites, respectively. The static 2‐PD of finger pulp was 4.5 mm (range: 4–6 mm). The wounds in the foot healed well, and no patient complained about the donor site. The patients’ information and the surgical data are summarized in Table 1.

**TABLE 1:** Patients’ information and the surgical data

No.	Age (years)	Sex	Cause	Size of defect (cm × cm)	Follow‐up (month)	2‐PD	Quick‐DASH scale	VSS (reconstructed site)	VSS (donor site)
1	23	M	Machine injury	1.5 × 3.0	6	4	6	4.1	3.9
2	18	M	Cut injury	3.5 × 5.5	30	4	9	3.9	4.6
3	36	M	Machine injury	1.5 × 4.5	39	6	10	4.5	4.3
4	34	M	Cut injury	2.5 × 4.0	24	4	5	4.2	4
5	34	M	Machine injury	2.0 × 4.0	12	4	7	3.8	3.5
6	33	M	Machine injury	3.5 × 4.0	26	5	6	4.6	4.2
7	23	F	Machine injury	3.0 × 4.0	34	5	5	4.1	3.9
8	23	M	Cut injury	4.5 × 3.5	30	4	11	3.9	3.9
9	22	M	Cut injury	2.5 × 4.5	18	4	4	4.4	4.4
10	22	M	Cut injury	1.5 × 3.5	9	6	5	3.9	4.5
11	20	F	Machine injury	2.5 × 2.5	10	6	6	3.7	4.7
12	19	M	Cut injury	3.5 × 3.5	16	4	4	3.9	3.6
13	32	M	Machine injury	1.5 × 5.0	19	4	9	3.8	3.6
14	30	M	Cut injury	2.5 × 4.5	25	5	11	3.5	3.8
15	27	F	Machine injury	4.0 × 4.0	28	4	10	4.4	3.4
16	27	M	Cut injury	3.5 × 3.5	22	4	7	4	3.4
17	26	M	Machine injury	2.5 × 4.0	30	4	8	3.9	4.2
18	24	M	Machine injury	1.5 × 4.5	33	5	5	3.8	4
19	54	F	Cut injury	3.0 × 4.0	27	4	9	4.1	3.9
20	43	M	Machine injury	2.5 × 3.0	35	4	5	3.8	4.1

Abbreviations: VSS, Vancouver Scar Scale.

### Case Reports

#### Case 1

**FIGURE 2:**
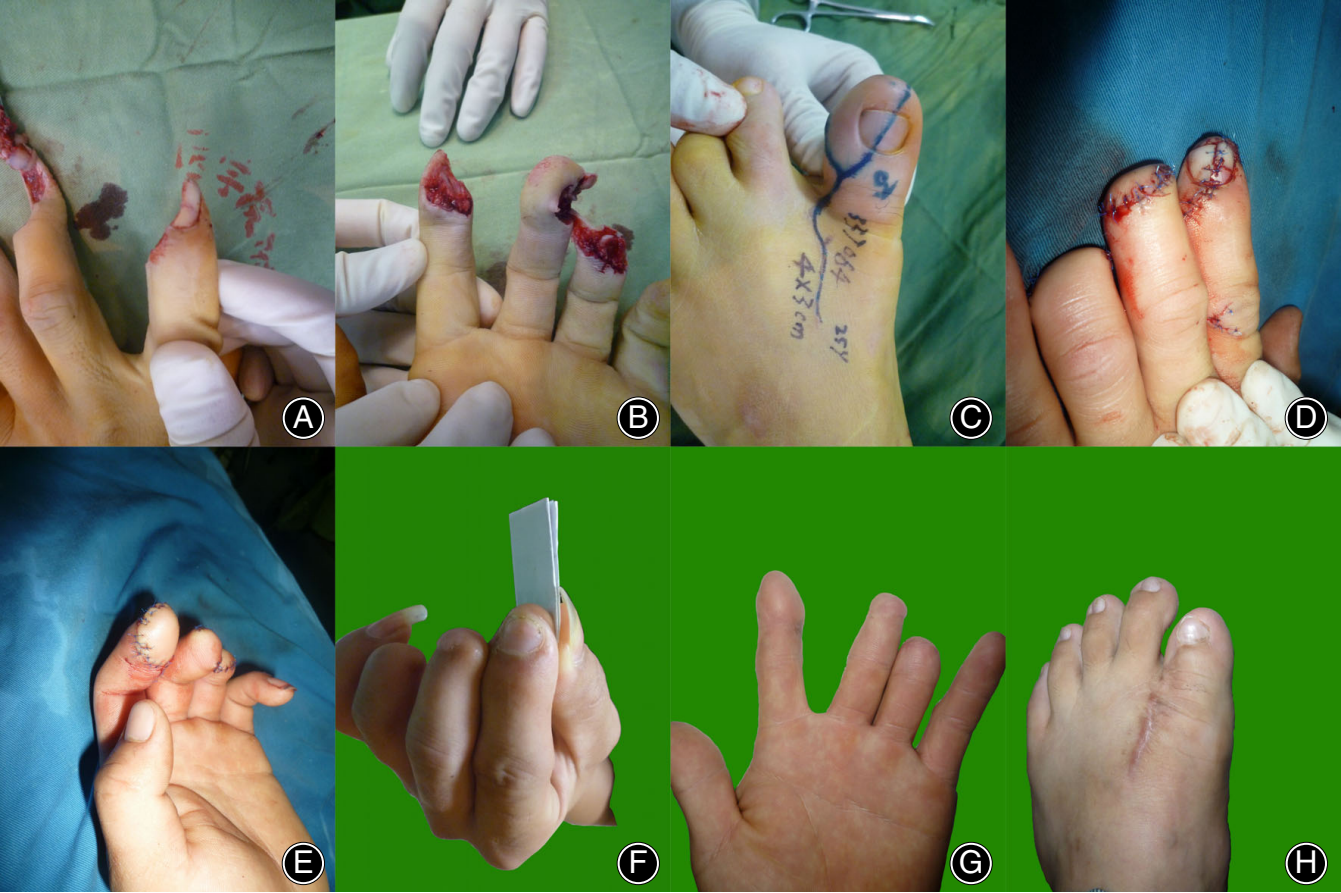
(A, B) A 25‐year‐old man presented with tissue loss on his left index finger from a machine accident. The serious injury to the distal middle and ring finger suggested no chance of maintaining the length of these fingers. (C) Preoperative design of the partial toenail flap from the ipsilateral great toe; (D, E) postoperative view of the new index finger after reconstruction; (F, G) the view and function of the index finger 1 year postoperatively; (H) postoperative 1‐year view of the donor great toe.

A 25‐year‐old man suffered skin tissue loss and a nail bed defect on the ulnar side of his index finger. His distal middle finger and ring finger had no possibility of being reconstructed after being crushed in a machine. After careful debridement, the defect was reconstructed with a 4.0 cm × 3.0 cm partial toenail flap from the ipsilateral great toe. The donor site was closed directly by trimming and shortening the bone. The flap survived, and the donor site wound healed well. No complications were encountered during the follow‐up period. We performed the static 2‐PD test 1 year after surgery, and the result was 5 mm. The patient was satisfied with the sensory function and aesthetic appearance at the 1‐year follow‐up. No morbidity of the donor site was observed (Figure 2).

#### Case 2

**Figure 3:**
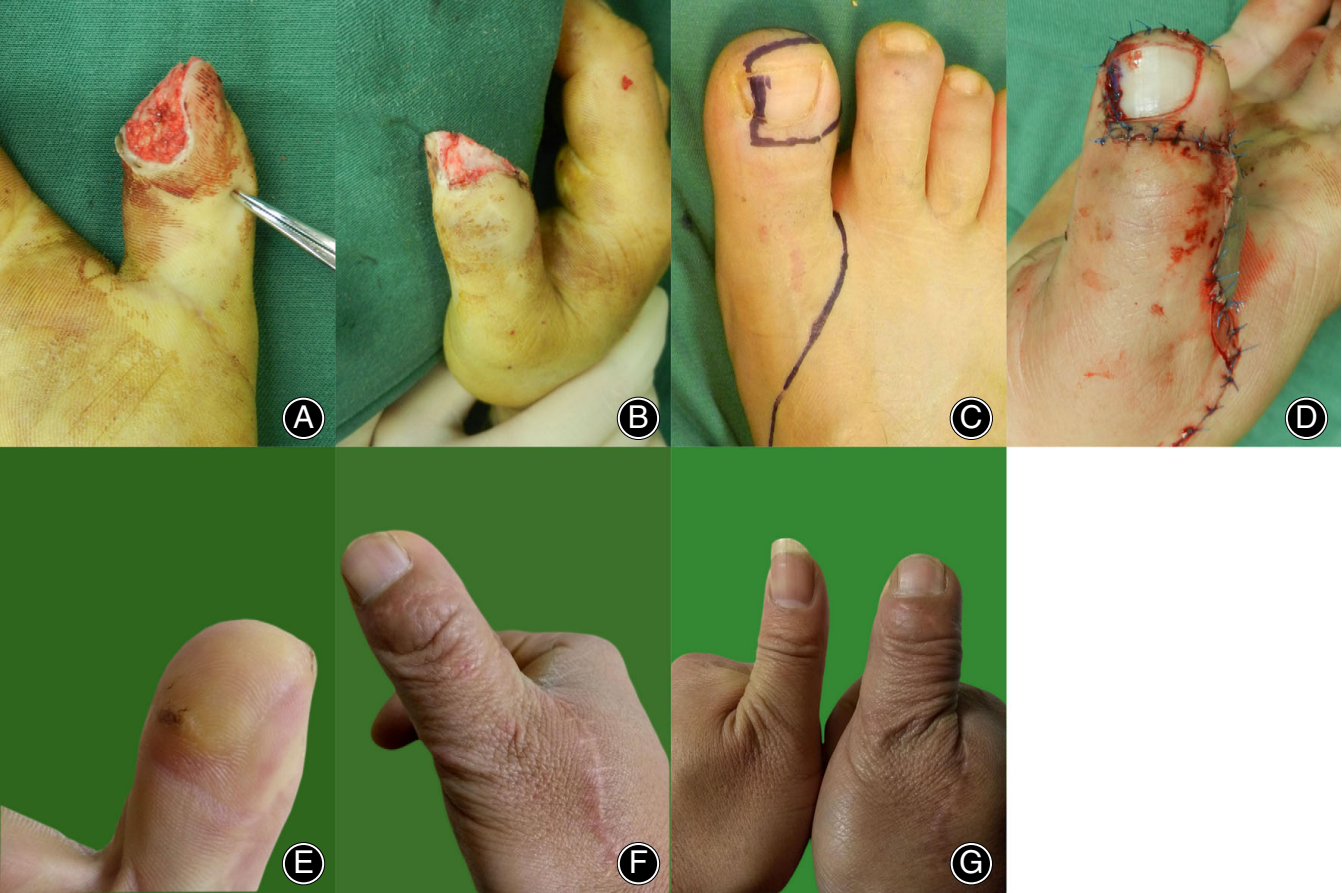
(A, B) A 39‐year‐old man sustained a laceration of the tip of his right thumb, with finger pulp and nail loss. (C) Preoperative design of the partial toenail flap from the ipsilateral great toe; (D) postoperative view after reconstructing the thumb pulp and the perionychium; (E–G) the repaired thumb at the 3‐year follow‐up.

A 39‐year‐old man lacerated the tip of his right thumb, with loss of finger pulp and perionychium. The distal phalanx was intact and exposed. After debridement and exploration of the defect wound, the defect size was 5.0 cm × 3.0 cm, and 3 cm of the radial common digit nerve was lost. We designed a 5.5 cm × 3.5 cm free partial toenail flap to rebuild the thumb tip. The donor site was covered by shortening the bone and revising the stump. The flap survived completely. The 24‐month follow‐up revealed satisfactory sensory and aesthetic results (Figure 3).

#### Case 3

**Figure 4:**
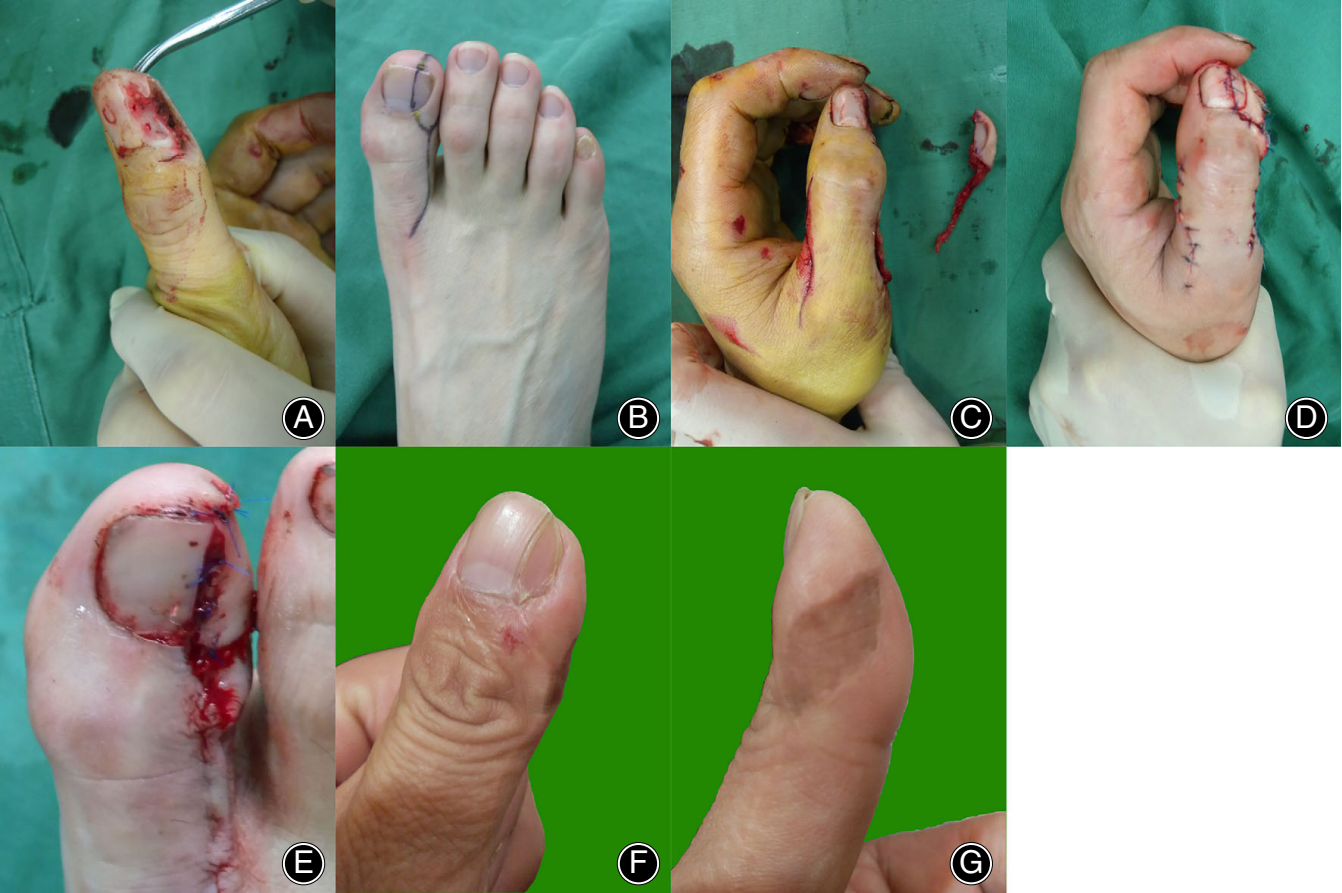
(A) A 39‐year‐old man suffered a soft tissue injury to his left thumb from a machine, losing the radial nail bed and pulp tissue. (B) Preoperative design of the partial toenail flap from the contralateral great toe; (C, D) Instant view of the fingertip reconstruction with a partial toenail flap. The lack of skin above the vascular pedicle was covered by a split skin graft, as we did not harvest the extra triangular skin. (E) The great toe pulp V‐Y advancement flap was used to cover the donor defect; (F, G) Postoperative 2‐year view of the repaired thumb. The skin graft with pigmentation on the radial and lateral sides of the thumb influenced the cosmetic outcome but did not affect movement.

A 39‐year‐old man suffered a soft tissue defect in his left thumb from a machine cut injury, with radial perionychium and partial pulp tissue loss. A 3.0 cm × 1.5 cm partial toenail flap was elevated to reconstruct the radial part of the thumb tip. Owing to the lack of triangular skin around the neurovascular pedicle, a portion of the pedicle was exposed, so a split‐thickness skin graft from the dorsal foot was applied to cover the defect. The toe pulp V‐Y advancement flap was used to cover the donor defect (Figure 4).

## Discussion

### Great Nail Flap Is Ideal for Treating Fingertip Loss

Skin tissue defects of the distal finger are common in patients with a hand injury. The nail is always simultaneously damaged with the finger pulp, and the tendon and bone can be exposed. Various methods, such as the V‐Y advancement flap,^6^ the digit neurovascular flap,^7^ the metacarpal artery flap,^8^ and the cross‐finger flap^9^ (all classical resurfacing treatments for a fingertip), have been widely used since microsurgical techniques were first developed. These flaps fail to reconstruct the nail in cases of multiple tissue defects and do not achieve cosmetic satisfaction, affecting the patient's pinching ability and confidence. In addition, sensitivity to the fingertip is not restored with these local flaps.

An ideal reconstructed distal finger requires adequate length, specialized digit‐like skin, good innervation, a stable well‐formed nail, and an aesthetic contour.^10^ Although Littler^11^ applied a digital neurovascular flap to reconstruct distal pulp defects of the thumb and index finger to achieve a finger‐like replacement, this approach sacrificed the main artery, the digit nerve, and the volar skin tissue of the healthy finger, which led to morbidity in the injured hand. Compound tissue transfer from a toe to a finger meets all the requirements for a newly rebuilt finger. Nicoladini^12^ first repaired a lost thumb using a pedicle great toe transfer. Dongyue^13^ successfully reconstructed a lost thumb with a free second toe transfer in 1966, and Cobbett^14^ applied the free great toe transplantation in 1968, extending the methods of utilizing the toe tissue transfer to reconstruct a finger or a portion of a finger. Foucher^15^ considered the foot an invaluable source of spare tissue for reconstructing finger portion defects after he performed different types of partial toe tissue transfers to rebuild finger tissue defects.

The nail is an irreplaceable finger structure that maintains stability when fingers pinch and grab. The nail also protects the fingertip and contributes to the tactile sensation needed to pick up small objects. The loss of a nail is a functional and cosmetic issue. A vascularized nail graft is the most reliable method for reconstructing total or partial nail defects.^16^ In the present study, all patients in this group presented with loss of the distal segment of a finger. If the defects were treated traditionally, the exposed distal phalanx would be covered using a skin flap transfer after the residual nail was removed, ignoring the importance of the nail in motion and aesthetics. Reconstructing the nail bed, the nail fold, the eponychium, the paronychium, and the hyponychium are essential to avoid a nail deformity after restoring the soft tissue. If the distal portion of a dominant finger (thumb, index finger, or middle finger) is lost, reconstructing the nail is as significant as reconstructing the distal finger pulp.

### The Operative Skill of Partial Toenail Flap Transfer

The structure of the distal finger pulp is different from other parts of the finger, as it is the “eye” of the hand, with increased sensitivity and cutaneous fat compared to other parts of the finger to effectively buffer external pressures. The distal finger pulp contains adipose and connective tissue. One end of the connective tissue is fixed in the periosteum of the phalanx, and the other end is in contact with the skin, which prevents the sliding of the volar skin. It plays a stabilizing role when the fingers grasp and pinch. The skin creases in the distal volar finger are called loops and whorls, which are unique to the finger, and the toe pulp has anti‐skid and anti‐wear functions. Based on these special anatomical structures, besides the flap transfer harvest from the healthy finger, the “anatomic reconstruction” of the distal finger pulp can only be transferred from the toe tissue due to the high similarity. While the soft tissue of the great toe is limited, compared to the second toe, it provides more tissue for designing a flap and closing the donor site. Partial toenail flap transfer not only achieves functional and aesthetic requirements but also minimizes mutilation of the foot.

The partial toenail flap is the segmented tissue of the great toe whose function remains after harvesting the flap. The great toe has an advantage compared to the second toe, as more composite flap designs can be employed using the great toe due to its adequate tissue supply. However, the dorsal skin in the partial flap is very small, making it difficult to carry the vein in the flap. Preserving a 1‐cm wide subcutaneous pedicle connected to the skin facilitates combining the vein in the flap. One or two dorsal cutaneous veins of the great toe run from the tibia side to the fibular side and from proximal to distal areas. If no vein was found on the tibia dorsal side, it was identified on the fibular side of the first web. At least two cutaneous veins were found in the dorsal metatarsophalangeal; however, only one slim vein could be carried in the flap. Extremely careful dissection should be performed in this tiny area. If any vessels are injured, the flap harvest will inevitably fail.

Although flap size is determined based on the size of a measured defect region, a lack of skin tissue around the vessel pedicle usually occurs after a partial toenail flap is sutured in the defect. The main reason is that the subcutaneous tissue, particularly the tissue around the vessel, is taken with the flap, increasing the size of the flap and tissue tension after resurfacing and postoperative swelling. A split‐thickness skin graft on the pedicle may resolve this problem, but scars often form. If extra triangular skin above the vessel in the flap is designed, this risk and further damage to the great toe are avoided (Figure 1). The extra skin will effectively relieve the tension of the most important part of the flap, where the neurovascular pedicle is located, provide reliable coverage of the vessel pedicle, and supply stable postoperative vascular circulation.

The total great toenail flap transfer functionally and cosmetically reconstructed the thumb defects. Complications at the donor site of the foot, such as skin necrosis or an infected skin graft, a contracted scar, and pain during weight bearing, were occasionally reported. Coverage of the great toe donor site remains a challenge for plastic surgeons not only because of the exposed bone and tendons but also because of the lack of locally available tissue.^17^ A partial toenail flap transfer is relatively less invasive to the great toe than an entire toenail flap transfer, which causes a smaller donor defect and more local tissue is maintained. The donor site was covered by a shortening revision of the distal phalange, the toe pulp V‐Y advancement flap, or a second toe neurovascular pedicle flap transfer to avoid the complications of a skin graft. The method of donor site coverage has improved, and outcomes have correspondingly improved.

### The Advantages and Limitations of Partial Toenail Flap

The partial toenail flap has a short pedicle, which is advantageous. First, the concern of encountering the first dorsal metatarsal artery (FDMA) of Gilbert III type is avoided. When the FDMA is of Gilbert III type, an additional plantar incision or vein graft is performed for a safe flap elevation. Second, The perforator flap proposed by Koshima was prone to vessel spasm and vessel kinking due to its long vascular pedicle, which affected the survival of the flap. In this study, a shorter vascular pedicle would decrease the risk of vessel spasm and vessel kinking,^16^ the length of vascular pedicle is much difference from the findings of Koshima.^16^ Normally, ligating a vessel at the metatarsophalangeal level is rather difficult for a safe anastomosis with the finger vessels, which is unnecessary to expose the tissue in the dorsal metatarsus. A short pedicle is minimally invasive and results in a shorter incision, a quicker surgery time, and a mini scar.

Many authors have defined the anatomical and meticulous restoration of the distal finger pulp and the nail as “aesthetic reconstruction.” However, the distal portions of the thumb, index finger, and middle finger have important functional roles. The indications for a partial nail flap transfer include good patient general condition and a surgeon with reliable microsurgical experience. Restoring the function of complex defects, such as loss of finger pulp and perionychium tissue and loss of the main finger (i.e., thumb, index finger, or middle finger), is the primary aim, and the aesthetic aim is of secondary significance for the anatomical reconstruction. In these case series, the partial toenail flap transfer is a worthy application as it enables patients to achieve near‐normal function and regain confidence; it is not a “decorative reconstruction.” However, this method has disadvantages of limited coverage size, the high microsurgical skill required, and the complex surgical process, and therefore might not be easy to popularize.

### Conclusion

It is undeniable that toe tissue transfer is the most appropriate treatment for a complex finger tissue defect. It is not a novel technique but is not widely used for indicated cases. The main reason is that a high degree of microsurgical skill is required. Second, morbidity of the foot could affect the postoperative life of the patient. Finally, there is insufficient recognition of the importance of repairing the distal fingertip, particularly in main fingers. Reconstructing the distal finger pulp and nail defects on the main fingers using partial toenail flap transfer is an ideal method to obtain anatomical restoration with good sensory function, a stable perionychium, and an excellent cosmetic appearance with natural curvature of the pulp, which helps the patient develop self‐confidence. The donor site does not require a skin graft, resulting in minimal morbidity.

## Author Contributions

LYC, ZJL and FYZ initiated the study design. FYZ is the principal investigator. ZJL is the programme coordinator. XZ and YYZ conceived the intervention and, with the support of GHX, is responsible for execution of the intervention, recruitment of participants and the administration of the study. LYC helps to coordinate data collection. GHX and PL help with the recruitment and, together with FYZ, provide clinical expertise.

## Ethics Statement

This study was performed in line with the principles of the Declaration of Helsinki. Approval for this case‐control investigation was obtained from the Second Affiliated Hospital and Yuying Children's Hospital of Wenzhou Medical University's Institutional Review Board.
